# Phosphorus transformations in plant-based and bio-waste materials induced by pyrolysis

**DOI:** 10.1007/s13280-017-0990-y

**Published:** 2017-11-20

**Authors:** James Stephen Robinson, Karen Baumann, Yongfeng Hu, Philipp Hagemann, Lutz Kebelmann, Peter Leinweber

**Affiliations:** 1 Department of Geography and Environmental Science, University of Reading, Reading, RG6 6AB UK; 2 Soil Science, Faculty for Agricultural and Environmental Sciences, University of Rostock, Justus-von-Liebig Weg 6, 18059 Rostock, Germany; 3 Canadian Light Source, Inc., University of Saskatchewan, 44 Innovation Boulevard, Saskatoon, SK S7N 2V3 Canada; 4 M.E.E. GmbH, Werkstraße 206, 19061 Schwerin, Germany

**Keywords:** Biochar, Bio-waste, Fertiliser, Phosphorus

## Abstract

Strategies are needed to increase the sustainability of phosphorus (P) fertiliser management in agriculture. This paper reports on the potential of pyrolysis treatment to recycle P from renewable materials previously regarded as wastes. The study used *K*-edge X-ray absorption near-edge structure (XANES) spectroscopy to examine chemical forms of P in the waste feedstock materials and corresponding biochars (pyrolysis at 480–500 °C) of four ligno-cellulosic, plant-based residues and five relatively P-rich livestock and water-treatment by-products, to acquire information on changes in potential P fertiliser value. Pyrolysis enriched P in the biochars by factors of 1.3–4.3, thus offering wide-ranging P fertiliser potential. XANES spectroscopy revealed hydroxyapatite (HAP) as one of the dominant chemical P compounds in the feedstocks, ranging from 14% (rice husks) to 98% (animal bone) of total P. For most materials, pyrolysis increased the proportion of HAP, and pyrophosphates were generated in several cases. These alterations possibly lead to diversity in the P solubility characteristics of the biochars if used as soil amendments; this is an important property of environmentally sound P fertilisers.

**Electronic supplementary material:**

The online version of this article (10.1007/s13280-017-0990-y) contains supplementary material, which is available to authorized users.

## Introduction

Inefficient methods of production, distribution and use of refined fertiliser phosphorus (P) for modern agriculture pose serious threats, not only to the aquatic environment, but also to global supplies of phosphate rock—the non-renewable raw material used for the vast majority of P fertiliser manufacture (Jarvie et al. 2015; Withers et al. 2015). Strategies for increasing the sustainability of P management in agriculture include recycling P from renewable materials previously regarded as wastes (Schroder 2005; Le Corre et al. 2009).

Current and potential sources of biomass for recycling P generally fall into one of the two categories: relatively P-enriched bio-waste (e.g. livestock manures and bone, municipal biosolids, anaerobic digestates and composts) or high C-containing, plant-based, ligno-cellulosic materials (e.g. wood, grain husks, nut shells) (Oenema et al. 2012). Compared with bio-wastes, the stable C content and high C:P and C:N ratios in ligno-cellulosic biomass render many of the latter unsuitable for fertiliser use. However, some plant-based residues (e.g. grains and grasses) have relatively high-mineral contents. For example, Amonette and Joseph (2009) report ash contents of rice husks as high as 24% by weight. These reports warrant further investigation into the P recycling potential of plant-based biomass.

In recent years, thermal treatment (e.g. pyrolysis) of biomass has emerged as an environmentally and economically sustainable waste management option (Hossain et al. 2011; Huang and Tang 2015). Benefits include the production of renewable and environmentally benign energy from locally available raw materials, and a reduction in the contents of water and environmental contaminants whilst retaining and concentrating the nutrient content of the solid by-product, biochar (Chan and Xu 2009; Wang et al. 2014). Biochars free from harmful levels of contaminants have long been added to agricultural land as safe and effective soil amendments (e.g. Hussain et al. 2016). Several studies have explained increases in the fertility of amended soils with respect to the carbon and fertiliser nutrient status of the biochar, both in relation to the feedstock type and pyrolysis conditions (e.g. Chan and Xu 2009; Wang et al. 2014). For example, significantly depending on the feedstock and solid residence time, low-to-moderate pyrolysis temperatures (400–500 °C) can favour the accumulation of potentially useful proportions of plant available P, among other macronutrients (Bourke et al. 2007; Chan and Xu 2009; Morales et al. 2013). However, relatively little is known about the effect of pyrolysis on the chemical forms of the P in the feedstock. Huang and Tang (2015) concluded that P speciation in biochars derived from a variety of treated sewage sludges was influenced by the pyrolysis conditions. Similar findings have been drawn from studies on the pyrolysis of plant and manure biochars (e.g. Zheng et al. 2013; Uchimiya and Hiradate 2014; Wang et al. 2014). Nonetheless, there is still a lack of fundamental knowledge regarding the extent to which P chemistry is changed by thermal treatment. This information underpins the potential P fertiliser value of the biochar and, therefore, would inform appropriate recommendations with respect to rate, frequency and timing of application to land.

Synchrotron-based X-ray absorption near-edge structure (XANES) spectroscopy has successfully been used to study P chemistry in bio-wastes and soils (Shober et al. 2006; Hesterberg 2010; Negassa et al. 2010). The P *K*-edge XANES can identify crystalline, poorly crystalline and amorphous forms of Ca-, Fe- and Al-bound P compounds (Hesterberg et al. 1999; Shober et al. 2006). Depending on data quality and representativeness of the P standards, the technique can also discern some organic P chemistry in environmental samples. However, the lack of distinct features in XANES spectra for many standards often hinders this ability (Peak et al. 2002; Toor et al. 2006).

There are only a few reports on the use of XANES spectroscopy to discern the transformation of P forms in biomass materials during pyrolysis (Zwetsloot et al. 2014; Bruun et al. 2017). The objectives of the present study were to use this analytical technique to systematically identify and quantify the major forms of P in a range of plant-based and bio-waste materials as well as in their biochars derived from pyrolysis treatment. The goal of the research was to provide a critical evaluation of pyrolysis as a treatment for enhancing and standardising the potential P fertiliser replacement value of diverse biomass materials.

## Materials and methods

### Sample collection and preparation

Nine biomass feedstocks and their derived biochars (18 samples in total) were selected for characterisation and P analysis. Four of the feedstocks were plant-based materials: wetland reed (*Phragmites australis* L.) [REED]; rice husks (*Oryza sativa* L.) [RICE]; acacia bark (tree species unknown) [ACAC]; and cocoa prunings (*Theobroma cacao* L.) [COCA]. The other five materials comprised bio-wastes: animal bone [BONE]; poultry manure [POUL]; pig slurry [PIG], and two anaerobically digested, municipal sewage sludges [SLDG and BIOS].

The reed samples were delivered by a local farming company in Mecklenburg-Vorpommern, Germany. To facilitate handling, the long fibres were shredded and pelletised. The rice husks were collected from Indonesia, and the acacia bark and cocoa prunings (young shoot cuttings) from small-holder, agro-forestry operations in Namibia and Ecuador, respectively. Defatted animal bone chips (C-quality, size 2–5 mm) were purchased from Sonac Ltd. Vuren, Netherlands. The poultry manure was obtained from a broiler chicken farm in Vilnius, Lithuania. The pig slurry, obtained from a swine unit in the Netherlands, was membrane-filtrated to a dry matter content of 97.8% (w/w). Samples of anaerobically digested, dewatered, and conditioned sewage sludges were collected from municipal wastewater treatment centres in Germany (Hohenburg, Bavaria, Germany) (SLDG) and the UK (Reading, UK) (BIOS). All materials were pyrolysed in industry-scale installations under N_2_ flow, between 480 and 500 °C, with solid residence times of between 10 and 20 min (M.E.E. EREKA and EPI Bioreactors, Germany and UK).

A portion of each feedstock and biochar sample was dried at 60 °C overnight and ground to pass a 0.5-mm screen using a stainless steel mill, in preparation for elemental analysis in triplicate and XANES spectroscopy.

### Elemental analysis

Total C and N were determined by a CN analyser (Vario EL III; Elementar Analysensysteme, Hanau, Germany), and total P, Ca, Mg, Al, Fe, Na and K by inductively coupled plasma optical emission spectroscopy (ICP OES) (JY 238 UL Trace, France) after microwave-assisted digestion in concentrated nitric acid and 30% hydrogen peroxide (USEPA, 1996).

### XANES spectroscopy

Twenty-eight reference standards were selected for the XANES spectroscopy, based on the prospect of their existence in the feedstock and/or biochar materials, and on their frequent publication in the relevant literature (Hesterberg 2010; Ingall et al. 2011). Mineral standards included Ca- (*n* = 4), Mg- (*n* = 3), Al- (*n* = 2), Fe- (*n* = 1), Na- (*n* = 2), K- (*n* = 4) and NH_4_-P (*n* = 2) forms; sorbed phase standards included Fe oxides (*n* = 1) and Al oxides (*n* = 1), and organic P standards included phospholipids (*n* = 2), nucleotide phosphates (*n* = 3), phytates (*n* = 1) and phosphonates (*n* = 2). See Appendix S1 for further information.

The P *K*-edge XANES spectra were recorded at the Canadian Light Source (CLS) in Saskatoon, Saskatchewan, Canada, on the Soft X-ray Micro-characterisation beamline (Hu et al. 2010). A small amount of sample was uniformly applied to P-free, double-sided carbon tape, attached to a stainless steel sample holder before insertion into the X-ray absorption chamber. Spectra for all samples and reference standards were recorded in total electron yield mode at photon energies between 2121 and 2200 eV for the P *K*-edge. Step sizes were 0.5 eV (2121–2140.5 eV), 0.15 eV (2140.65–2162.4 eV) and 0.5 eV (2162.5–2200 eV) and the dwell time 4 s (samples) and 1 s (reference standards). For each sample, at least two scans were recorded and the sample holder position was changed after each scan so that the beam always hit a ‘fresh’ sample spot. Subsequent data treatment and evaluation such as spectra averaging, background correction and normalisation to an edge-step of one were done using the ATHENA software package (Ravel and Newville 2005). The spectra were normalised by fitting a cubic spline function to the post-edge and a linear baseline to the pre-edge region. For consistency in the subsequent linear combination fitting (LCF) analysis, all spectra (both for the references and samples) were normalised within the same range. The LCF, in the range between 2140 and 2190 eV, was performed on the samples using all possible binary to quaternary combinations of the above 31 P reference standards. No energy shift was allowed during the fit. Goodness of fit was evaluated using the residual factor (R-factor) generated by the LCF tool in ATHENA.

## Results and discussion

### Elemental composition of materials

#### Feedstocks

The reeds, rice husks, and cocoa prunings have mineral contents substantially higher than those of the woody acacia material (Table 1). The relatively sparse mineral concentrations in the acacia bark are typical of woody feedstock. Total P, by weight, in the plant-based materials ranges from 0.3 (acacia bark) to 1.1 g kg^−1^ (cocoa prunings). The P contents of the reed, rice, and cocoa samples are similar to previous reports (e.g. Zheng et al. 2013; Gao et al. 2015); the relatively high P content of the cocoa prunings possibly reflects recent P fertiliser history. Compared with the plant materials, the lower C and predominantly higher mineral contents of the bio-waste feedstocks reflect the high-mineral diets of animals and humans. Total P ranges almost by an order of magnitude, from 13 to 104 g kg^−1^, decreasing in the order: bone > BIOS sewage sludge > pig slurry > SLDG sewage sludge > poultry manure (Table 1).

**Table 1:** Elemental composition (g kg^−1^) of the biomass feedstocks and their derived biochars

Sample	C	N	P	K	Ca	Mg	Fe	Al
Plant-based								
REED	413	9.9	0.7	7.3	3.6	0.9	0.2	0.1
REED-B*	546	14.6	3.0	24.2	13.8	3.3	6.6	1.2
RICE	381	4.0	0.6	1.9	1.1	0.4	0.2	0.2
RICE-B	495	7.7	1.6	4.6	3.7	1.2	2.1	1.2
ACAC	496	8.9	0.3	2.9	24.7	2.1	0.4	0.1
ACAC-B	681	10.6	0.4	1.6	27.2	2.1	0.5	0.5
COCA	453	5.3	1.1	6.3	2.6	1.6	0.8	0.1
COCA-B	633	6.5	2.4	19.2	6.0	2.7	2.2	1.4
Bio-waste								
BONE	178	50.5	104	1.3	195	4.1	0.1	0.0
BONE-B	104	18.6	130	1.9	238	5.5	0.1	0.1
POUL	347	44.6	12.6	17.9	60.1	6.4	1.0	0.1
POUL-B	311	28.1	35.3	49.1	132	17.5	2.2	0.4
PIG	378	26.9	20.4	13.7	25.1	13.1	5.6	0.9
PIG-B	381	22.8	51.1	30.3	56.7	33.0	15.9	2.5
SLDG	342	53.4	15.8	3.8	27.1	6.0	11.0	17.7
SLDG-B	226	17.8	28.6	6.4	48.9	9.8	20.7	26.4
BIOS	399	34.5	24.3	1.3	19.9	3.3	4.3	1.9
BIOS-B	382	19.0	60.0	3.4	40.9	9.5	11.6	9.9

-B* denotes biochar derived from pyrolysis of the feedstock

#### Biochars

Generally, the C and mineral contents of the biochars follow approximately the same orders as those for the feedstocks (Table 1); this common trend reflects the close dependency of biochar properties on the composition of the feedstock (Verheijen et al. 2010). Relatively high-mineral containing grass, grain husks, straw residues and manures generally produce biochar with high ash contents, in contrast to that from woody feedstocks (Demirbas 2004). For instance, some manure (e.g. chicken litter) and sewage sludge (e.g. Al-treated biosolids) biochars can contain as much as 50 and 5% (by weight) ash and P, respectively (Amonette and Joseph 2009; Wang et al. 2012). For all of the plant-based materials, the C content of biochar is higher than that of the source feedstock (Table 1). This increase in concentration is attributed to pyrolysis removing a considerable mass of material in the form of volatiles (e.g. hydrocarbons, H_2_, CO and CO_2_), and leaving behind a stable matrix of largely aromatic C (Baldock and Smernik 2002; Demirbas 2004). During thermal degradation of the plant biomass, N content vaporises at relatively low temperatures, after peaking at around 300 °C (Baldock and Smernik 2002). Conversely, due to increased stability, P, S and most metals vaporise at temperatures that are considerably higher; e.g. P at around 1000 °C (Zhang et al. 2012). Similar processes occur during the pyrolysis of the bio-waste materials; however, the higher proportions of labile C and N in bio-wastes relative to those in plants explain the thermally induced decreases in these parameters (Table 1). For all materials, the differential vaporisation of elements during pyrolysis explains why P is retained and becomes concentrated in the biochars. Indeed, such was the concentrating effect in the reed, rice and cocoa biochars, that the total P contents of these three materials reached values (1.6–3 g kg^−1^) comparable with those of green waste compost (Chan and Xu 2009).

### Phosphorus K-edge XANES analysis of materials

**Fig. 1:**
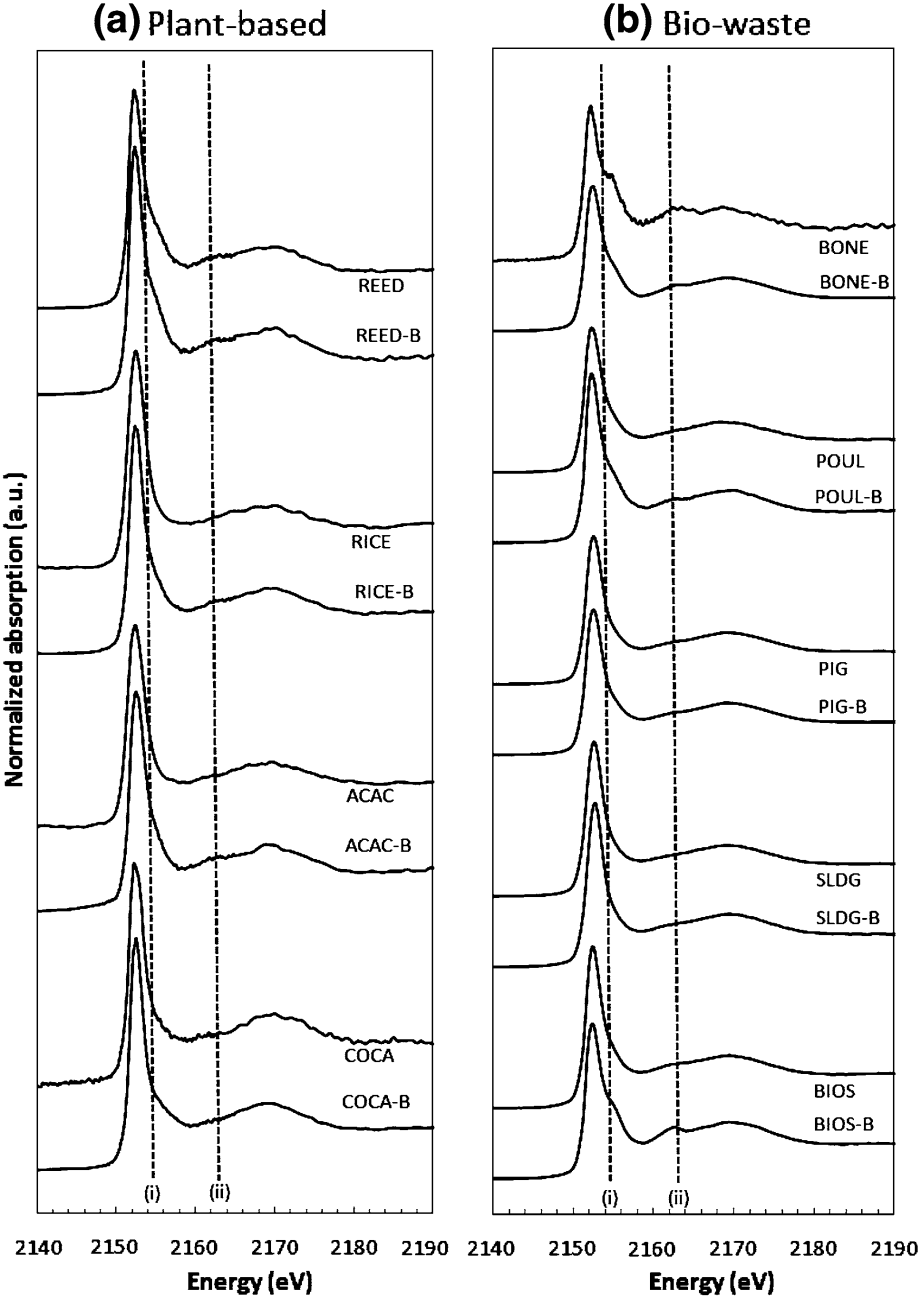
Stacked normalised P *K*-edge XANES spectra of the biomass feedstocks and their derived biochars (-B): **a** plant-based and **b** bio-waste materials. Sample identification is explained in the “Materials and methods” section

The normalised P *K*-edge XANES spectra are shown in Fig. 1 for the plant-based and bio-waste materials and for their derived biochars. Generally, the signal-to-noise ratios in the spectra for bio-wastes were higher than those for the plant materials, consistent with the higher P contents of the former (Table 1). Pyrolysis did not appear to alter the signal-to-noise ratio for any of the materials except, perhaps, for the slight improvement in the bone (Fig. 1b). All spectra shared a similar shape, with a dominant narrow peak at around 2152.5 eV (the P absorption edge) followed by a much broader peak at around 2169 eV (oxygen oscillation). Descriptions of diagnostic features of P *K*-edge XANES spectra for all phosphate standards used in this study can be found in Hesterberg (2010) and Ingall et al. (2011). Overall, XANES spectra for only 7 of the 28 standards evaluated yielded the best linear combination fits to the spectra of the samples (Table 2).

**Table 2:** Relative proportions of phosphorus standards that provide the best fit to P *K*-edge XANES spectra of the biomass feedstocks and their derived biochars according to linear combination fitting. Values < 5% are deemed unreliable

Sample	Hydroxyapatite	AlPO_4_·xH_2_O	FePO_4_·xH_2_O	Mg_2_O_7_P_2_	Na_2_HPO_4_·xH_2_O	P sorbed to Fe oxides	ATP (Na salt)	Goodness-of-fit (*R* factor)
Plant-based								
REED	39	–	–	–	–	26	35	0.010
REED-B*	53	–	–	–	–	41	6	0.013
RICE	14	–	–	–	–	42	44	0.014
RICE-B	28	–	–	7	–	38	27	0.008
ACAC	19	–	–	–	–	27	54	0.011
ACAC-B	37	–	–	–	–	47	16	0.004
COCA	30	–	–	–	–	51	19	0.014
COCA-B	24	–	–	38	–	31	7	0.009
Bio-waste								
BONE	98	–	–	–	–	2	–	0.011
BONE-B	56	–	–	44	–	–	–	0.004
POUL	34	–	–	–	–	14	52	0.003
POUL-B	48	–	–	17	–	16	19	0.006
PIG	23	–	–	–	30	30	17	0.001
PIG-B	26	–	–	–	33	28	13	0.001
SLDG	25	4	–	–	–	32	39	0.002
SLDG-B	30	15	–	–	–	38	17	0.002
BIOS	36	–	–	1	–	39	24	0.002
BIOS-B	61	–	19	20	–	–	–	0.001

-B* denotes biochar derived from pyrolysis of the feedstock

The range of R-factors in Table 2 was comparable with those of other recent publications with LCF of P *K*-edge XANES spectra (e.g. 0.0009–0.123 in Kruse et al. 2010; 0.0006–0.022 in Prietzel et al. 2016) based on different P-reference compounds and recorded at different beamlines. However, P standards that accounted for less than 5% of total P are deemed unreliable, reflecting the limitations of the XANES and LCF procedures. For each material, comparisons are shown between the measured and LCF-derived spectra (Fig. S1).

#### Plant-based materials

In general, the combination of P standards yielding the best LCFs for the plant materials were hydroxyapatite (HAP), P sorbed to Fe oxides, and ATP (Na salt) (Table 2). Hydroxyapatite was identified by LCF as the dominant P form in the reed. The spectra of the reed feedstock and corresponding biochar displayed a prominent post-edge shoulder at around 2154.5 eV [Fig. 1a: line (i)], characterising calcium phosphates. Whilst evident, the equivalent feature is weaker for the other plant spectra, reflecting lower contents of HAP (Table 2). The prominence of the HAP reflects the fundamental importance of Ca in plant cellular metabolism, whereby Ca-bearing minerals comprise as much as 50% of known biominerals, with approximately half in the form of calcium phosphates (Weiner and Dove 2003). Weiner and Dove discuss in detail the abundance of carbonate-substituted HAP in biological material, but note that there is no evidence for the biological formation of the non-carbonated form. On the contrary, a combination of X-ray powder diffraction (XRD), thermal analysis and Fourier transform infrared spectroscopy (FITR) has identified non-substituted HAP as a significant inorganic constituent of some plant species (Shaltout et al. 2011). The LCF was unable to distinguish between substituted and non-substituted forms of HAP in the current study.

Among the plant-based feedstocks and biochars, there was no clear suggestion of a pre-edge peak (around 2149 eV) in the P *K*-edge spectra (Fig. 1a); this points towards a lack of evidence for precipitation of non-crystalline or crystalline Fe phosphate minerals (Hesterberg et al. 1999; Shober et al. 2006). Nonetheless, Fe-bearing minerals comprise approximately 40% of biominerals (Weiner and Dove 2003); many of these exist as a variety of oxides, hydroxides and hydrous oxides—all characterised by a strong affinity to sorb P. A further possible explanation for the Fe–P phases is contamination of the plant materials with attached soil or dust particles.

The LCF indicated that ATP (Na salt) was the only organic P form in the plant-based materials and their biochars (Table 2). Whilst it is likely that ATP does exist in the feedstocks, it is well known that the XANES spectra of organic P reference compounds lack distinguishable pre- or post-edge features (Peak et al. 2002; Shober et al. 2006); this renders organic P speciation difficult (Toor et al. 2006). As such, it is likely that the ATP is represented in unrealistically high quantities in the feedstocks and probably comprises additional organic polyphosphates also, such as phytic and nucleic acids.

As expected, the proportion of total P as organic P (albeit poorly characterised) in all four plant-based samples decreased following pyrolysis. This loss at moderate pyrolysis temperatures can be attributed to the cleaving of organic P bonds in the feedstocks, and has been demonstrated by others, using ^31^P-NMR spectroscopy; for example, Uchimiya and Hiradate (2014) reported a major decline in phytate from residues of cotton seed hull upon pyrolysis at around 350 °C. It is speculated that the persistence of the ATP signal following pyrolysis is due in part to the formation of inorganic polyphosphates. For three of the four plant materials (reed, rice, and acacia), the proportions of HAP increased markedly with pyrolysis; indeed, according to the LCF results, estimates in the biochars of rice and acacia doubled those in the corresponding feedstocks (Table 2). While some workers have claimed that the removal of organic matter by combustion (e.g. calcination) is necessary to thermally form crystalline HAP in plant biomass, the current findings appear to agree with Zheng et al. (2013) who reported increases in crystalline forms of Ca- and Mg phosphates following pyrolysis.

In the case of the cocoa, pyrolysis appears to shift the P chemistry towards pyrophosphates (Mg_2_O_7_P_2_), at the expense of all other dominant forms (Table 2). A smaller increase is observed for the rice. Previous studies on the pyrolysis of plant biomass at temperatures between 350 and 650 °C have observed a dominance of pyrophosphate in the biochar; this is thought to originate from the degradation of phytates (Uchimiya 2014; Degenstein et al. 2015).

#### Bio-waste materials

The LCF results indicated that HAP, P sorbed to Fe oxides, and organic P (represented by ATP) were dominant in all but one (bone chips) of the five bio-waste feedstocks (Table 2). In addition, the pig slurry recorded a substantial proportion (30%) of hydrated soluble P salt (Na_2_HPO_4_·xH_2_O) (Table 2).

According to the LCF, as much as 98% of the P in the bone chips exists as HAP (Table 2), and is characterised by the permanent post-edge shoulder around 2154.5 eV (line i), as shown in Fig. 1b. An additional, well-defined calcium phosphate feature appears around 2163 eV (line ii). Pyrolysis of the bone appeared to transform almost half of the HAP to metal pyrophosphate. Given the dominance of Ca in the bone, it is possible that a portion of total P in the bone char that was fit as Mg_2_O_7_P_2_ (44%) was actually Ca_2_O_7_P_2_. Based on the LCF of P *K*-edge XANES spectroscopic characterisation of rendered bones sampled from the poultry industry, Zwetsloot et al. (2014) reported lower proportions of HAP (60% of total P) than those estimated here. Moreover, these workers observed marked increases in HAP (to as much as 93%) following pyrolysis at temperatures (350–550 °C) similar to those applied in the current work. Further comparisons between the studies are difficult, primarily due to the absence of pyrophosphate standards in Zwetsloot et al. (2014), and also the possibility that the crystallinity of the HAP standards differed between the studies. The LCF indicated that ATP accounted for as much as 52% of total P in the poultry manure (Table 2). As we discussed previously, it is likely that the P estimated as ATP includes contributions from a broad range of P polymers. In the case of non-ruminant (e.g. poultry and pigs) manures, the dominant form is probably phytate. Phytic acid composes 60–80% of total P in cereals, oilseeds and legumes that are commonly used in livestock feed. As such, owing to its poor digestibility, phytic acid can account for as much as 60% of total P in manures, with smaller amounts of diester P compounds; e.g. phospholipids and DNA (Shober et al. 2006; Uchimiya and Hiradate 2014). Similar to its decline in the plant-based materials, the size of the organic P pool in the poultry manure decreased markedly with pyrolysis (Table 2), with the reduced ATP signal (19% of total P) probably comprising inorganic forms of polyphosphate. Consistent with the ^31^P-NMR findings of Uchimiya and Hiradate (2014), the majority of the mineralised P at relatively low pyrolysis temperatures transformed into inorganic orthophosphate and pyrophosphate; dominant inorganic compounds appearing in the poultry manure biochar included HAP and inorganic Mg_2_O_7_P_2_ (Table 2). According to the LCF, P sorbed to Fe oxides maintained a significant presence, increasing slightly from 14 to 16% of total P following pyrolysis (Table 2). However, such small variations could be assessments of noise in the analysis rather than real changes, and should be regarded with caution, especially in view of the relatively poor fit (*R*-factor = 0.006) for the poultry manure biochar compared with its feedstock (*R*-factor = 0.003) (Table 2; Fig. S1).

In spite of net increases in aromatic C at the expense of aliphatic groups, this region of pyrolysis temperature is known to produce labile organic matter that can condense on the biochar surface (Lu et al. 2013); this organic matter contains certain hydroxyl groups that may coordinate with Ca and favour the formation of HAP. It is possible that the aromatic C fraction stabilises the HAP on the biochar surface as nano-crystals (Uchimiya 2014). Possible mechanisms for the appearance of pyrophosphate in the poultry manure biochar include the general fragmentation of long-chain polyphosphates into shorter chains, or the thermal degradation of phytate to produce orthophosphate that thermally dehydrates to produce pyrophosphate. Regardless of the source, it is possible that the pyrophosphate becomes stabilised by forming complexes on the biochar surface (Uchimiya and Hiradate 2014).

In addition to the substantial amounts of HAP (23%) and polymerised (17%) P in the pig slurry, soluble Na_2_HPO_4_·xH_2_O accounted for as much as 30% of total P (Table 2). Similarly, previous workers have reported relatively high concentrations of water extractable P in pig slurry (Leinweber 1996). Compared with all of the other materials studied, the pig slurry exhibited relatively little change in P chemical forms due to pyrolysis. The minor, apparent decreases in the proportions of sorbed P (2%) and organically bound P (4%) were matched almost by increases in HAP and Na_2_HPO_4_·xH_2_O (3%). As noted earlier, such small shifts are probably assessments of noise, in spite of the relatively confident values for goodness-of-fit (R-factor = 0.001) (Table 2; Fig. S1). As previously speculated, the ATP fraction possibly conceals thermal transformations from organic to inorganic polyphosphates. Moreover, it is also possible that drying the pig slurry at 60 °C transformed the P into forms that remained relatively resistant to the subsequent pyrolysis.

The two sewage sludges (SLDG and BIOS) were dominated, but to different extents, by HAP, P sorbed to Fe oxides and ATP. A minority (< 5%) of P in the SLDG was precipitated as hydrated AlPO_4_ (Table 2). These dissimilarities occur in spite of the biosolids having received similar sludge treatment processes, and possibly reflect differences in composition between the two source wastewaters. The presence of polyphosphates in both sludges is explained by the primary process for removing soluble P (and N and K) from municipal sewage; prokaryotic accumulation is a microbiological process that accumulates the P in organic polyphosphate compounds as intracellular granules (Mehta et al. 2015). Pyrolysis of both sludges yields an overall increase in metal-associated P at the expense of organic P, but the transformations differ between the two materials. The large increase in Ca-P in the BIOS biochar, relative to that in the SLDG (Table 2) is evident in the spectrum at around 2163 eV (line ii), as shown in Fig. 1b. According to the LCF analysis, there is no longer a presence of adsorbed P on Fe oxides but, instead a marked appearance of FePO_4_·xH_2_O (Table 2). However, the absence of a clear, pre-edge peak in the XANES spectra questions the validity of this analysis. The apparent total loss of organic P (from 24% of total P) in the BIOS feedstock is almost matched by the appearance of pyrophosphate in the biochar (20%), indicating that the Mg_2_O_7_P_2_ was derived mostly from the transformation of intracellular, long-chain polyphosphates. In the case of the SLDG, no new P compounds are introduced by pyrolysis; instead, as reflected in the similarity between the two spectra, all three mineral P forms simply increase, albeit by different extents (Table 2, Fig. 1b).

### Implications for enhancing and standardising P fertiliser value

In general, pyrolysis of the feedstocks at the moderate temperature of 480–500 °C effected a transformation of the P content among seven dominant forms (Table 2). In most cases (rice, acacia, cocoa, bone, poultry manure, and both sewage sludges), new P compounds were introduced in the derived biochar; all but one (BIOS sludge) of these seven instances exhibited an increase in the number of P compounds.

In three of the four plant materials (reed, rice and acacia), pyrolysis increased the contribution of crystalline HAP to the total P pool, probably at the expense of organic P forms. Based on XRD techniques, previous workers also have reported an increase in crystallisation reactions involving Ca (and Mg) phosphates in plant-based materials around 500 °C (e.g. Zheng et al. 2013). Whilst HAP has viable, slow-release P fertiliser potential in acid soils, its actual dissolution would likely depend on its association with the biochar C. Relative to other functional groups, the proportion of aromatic C in pyrolysed biomass increases markedly at around 400 °C (Keiluweit et al. 2010). This aromatic carbon fraction incorporates significant proportions of P (and N and S) within its ring structure as heteroatoms (Bourke et al. 2007). In this state, soil chemical properties that characteristically drive the dissolution of sparingly soluble Ca phosphates (acid pH, ionic exchange and sorption capacities) would have limited physical access to the P. On the other hand, discrete, nano-sized, Ca–P-containing crystals have been observed on biochar surfaces, where they might be exposed to P dissolution and desorption reactions in the soil. Moreover, at pyrolysis temperatures between 400 and 600 °C, fresh carboxyl and hydroxyl C can form in addition to aromatic molecules; through complex reactions with Ca and Mg, these functional groups can prevent the formation of less soluble P minerals. A further factor that can influence HAP solubility is the degree of carbonate substitution in the crystal lattice (Pan and Darvell 2010); however, the XANES spectroscopy was unable to discern any such transformations in the current study.

In the case of the P-enriched bio-wastes, with the exception of the bone chips, pyrolysis produced larger proportions of HAP (Table 2). Perhaps surprisingly for P-enriched bio-wastes, moderately soluble forms of Ca phosphate (e.g. monetite, tricalcium phosphate) were not identified either in the feedstocks of their biochars. Nonetheless, similar to the case for most of the plant-based feedstocks, the thermally induced shift in P from organic forms to HAP (except bone) possibly represents an increase in slow-release fertiliser value in respect to acid soils. However, the aforementioned caveats concerning the protection of P minerals through interactions with aromatic C in plant-derived biochars also apply to bio-wastes (Uchimiya 2014; Uchimiya and Hiradate 2014).

It is likely that the appearance of pyrophosphates in biochars originated from transformations of phytic acids and polyphosphates in the feedstocks, or dehydration of high levels of HAP in the case of the bone. It is important to note that some metal pyrophosphates have an order of magnitude higher water solubility than phytates and, therefore, present potential fertiliser P value. The substantial, thermally induced shifts to pyrophosphates in the bone chips, poultry manure and BIOS sewage sludge possibly equate to increases in fertiliser P value for these materials. However, this difference might be tempered by the formation also of less soluble inorganic polyphosphate compounds (inferred from the persistence of ATP signals following pyrolysis), and through complex interactions between pyrophosphates and the ash or aromatic C contents generated during pyrolysis (Uchimiya and Hiradate 2014). As such, research is needed on the relative solubility of the HAP, metal pyrophosphate and polyphosphate components of biochars in different soils.

Following pyrolysis of the plant feedstocks, large but variable proportions of P remained or became sorbed to Fe oxides (except cocoa) (Table 2). The association of P with Fe oxides in plant-derived biochars produced by moderate pyrolysis temperatures is supported by the chemical extraction data of Uchimiya and Hiradate (2014). In spite of the comparatively low P concentrations in the plant biochars, the P sorbed to Fe oxides may pose a potentially valuable source of fertiliser P. With regard to the pig slurry and both sludges, the unusually high proportion of P sorbed to Fe oxides could make a substantial contribution to these materials’ fertiliser P value, whereas, as discussed previously, pyrolysis of the BIOS sludge appears to shift this P into precipitates of ortho- and pyro-phosphates.

## Conclusions

Generally, ligno-cellulosic feedstock and its derived biochar are often regarded as unsuitable for P fertiliser use owing to limited P content and a high C:P ratio. However, the total P contents of three of the plant feedstock biochars (reed, rice and cocoa) were similar to that of green waste compost. Moreover, in an acid soil, the dominant form of P in the biochar (HAP) is likely to be more soluble than that found in green waste compost as well as in the biochar feedstock (phytate). Hence, for P-deficient, acid soils in low-input agriculture, some ligno-cellulosic biochars could be considered as alternative P fertilisers to supplement their other beneficial effects as soil amendments, for example, in respect to soil pH, cation exchange and water holding capacities. Pyrolysis of the P-enriched bio-wastes caused marked and variable shifts in the relative proportions of largely insoluble P compounds (HAP, phytates and pyrophosphates). It is advised that the current observations using XANES should benefit from additional, complementary spectroscopic techniques (e.g. ^31^P-NMR) and solubility tests (e.g. sequential P fractionation), prior to direct investigations into the materials’ relative P fertiliser values through standardised plant bioassays.

## Electronic supplementary material

Below is the link to the electronic supplementary material.

Supplementary material 1 (PDF 591 kb)
