# Supplementary material for: Phosphorus transformations in plant-based and bio-waste materials induced by pyrolysis
Source: Ambio. 2017 Nov 20;47(Suppl 1):73–82. doi: 10.1007/s13280-017-0990-y (PMC5722748; doi:10.1007/s13280-017-0990-y)
Supplement: Supplementary file 1 — Supplementary material 1 (PDF 591 kb) [file 13280_2017_990_MOESM1_ESM.pdf]

Ambio

Electronic Supplementary Material

Title: **Phosphorus transformations in plant-based and bio-waste materials induced by pyrolysis**

Authors: James Stephen Robinson, Karen Baumann, Yongfeng Hu, Philipp Hagemann, Lutz Kebelmann, Peter Leinweber

Appendix S1. Reference standards selected for the P *K-edge* XANES spectroscopy. Chemicals were purchased from Sigma-Aldrich Laborchemikalien GmbH, D-30926 Seelze, Germany.

Dicalcium phosphate: $CaHPO_4$, CAS 7757-93-9

Brushite: $CaHPO_4.2H_2O$, CAS 7789-77-7

Calcium dihydrogen phosphate: $Ca(H_2PO_4)_2.2H_2O$; CAS 7757-93-9

Hydroxyapatite: $Ca_5H(PO_4)_3.2.5H_2O$; CAS 1306-06-5

Magnesium phosphate: $MgHPO_4.3H_2O$, CAS 7782-75-4

Tri-magnesium phosphate: $Mg_3(PO_4)_2.8H_2O$, CAS 13446-23-6

Magnesium pyrophosphate: $Mg_2O_7P_2$, CAS 13446-24-7

Aluminium phosphate: $AlPO_4.xH_2O$, CAS 66905-65-5

Aluminium metaphosphate: $Al(PO_3)_3$, CAS 13776-88-0

Iron (III) phosphate tetrahydrate: $FePO_4.4H_2O$, CAS 31096-47-6

Sodium dihydrogen phosphate: NaH_2PO_4, CAS 7558-80-7

Disodium phosphate: $Na_2HPO_4.2H_2O$, CAS 10028-24-7

Potassium dihydrogen phosphate: KH_2PO_4, CAS 7778-77-0

Dipotassium phosphate: $K_2HPO_4.3H_2O$, CAS 16788-57-1

Tripotassium phosphate: K_3PO_4, CAS 7778-53-2

Potassium pyrophosphate: $K_4P_2O_7$, CAS 7320-34-5

Ammonium dihydrogen phosphate: $NH_4H_2PO_4$, CAS 7783-28-0

Di-ammonium phosphate $(NH_4)_2HPO_4$, CAS 7722-76-1

P sorbed to Fe oxides: Goethite (A16267, purchased from Alfa Aesar, Thermo Fisher, Kandel GmbH, D 76057, Karlsruhe/Germany), and 2-line-ferrihydrite

(prepared according to the method of Schwertmann and Cornell, 2008) were equilibrated with KH_2PO_4 do achieve a desired P-concentration of 2000 µmol l^{-1} KH_2PO_4 with 0.01 M $CaCl_2$-background electrolyte solution and initial pH of 6. The remaining solid matter was dried for 24 h at 40°C and stored in a desiccator.

P sorbed to Al oxides: A synthetic and commercially available gibbsite (analytical grade, Merck Millipore, D 64293 Darmstadt/Germany) was loaded with P as described for the Fe-oxides.

Phosphorylethanolamine: CAS 39382-08-6

Asolectin: MFCD00146015

Adenosine 5′-monophosphate disodium salt: CAS 4578-31-8

Adenosine 5′-diphosphate disodium salt: CAS 16178-48-6

Adenosine 5′-triphosphate disodium salt: CAS 34369-07-8

Phytic acid sodium salt hydrate: CAS 14306-25-3

2-aminoethylphosphonic acid: CAS 2041-14-7

Phenylphosphonic acid: CAS 1571-33-1

References:

Schwertmann U, Cornell RM: Iron Oxides in the Laboratory. Preparation and Characterization, Hoboken: Wiley-VCH, 2008.

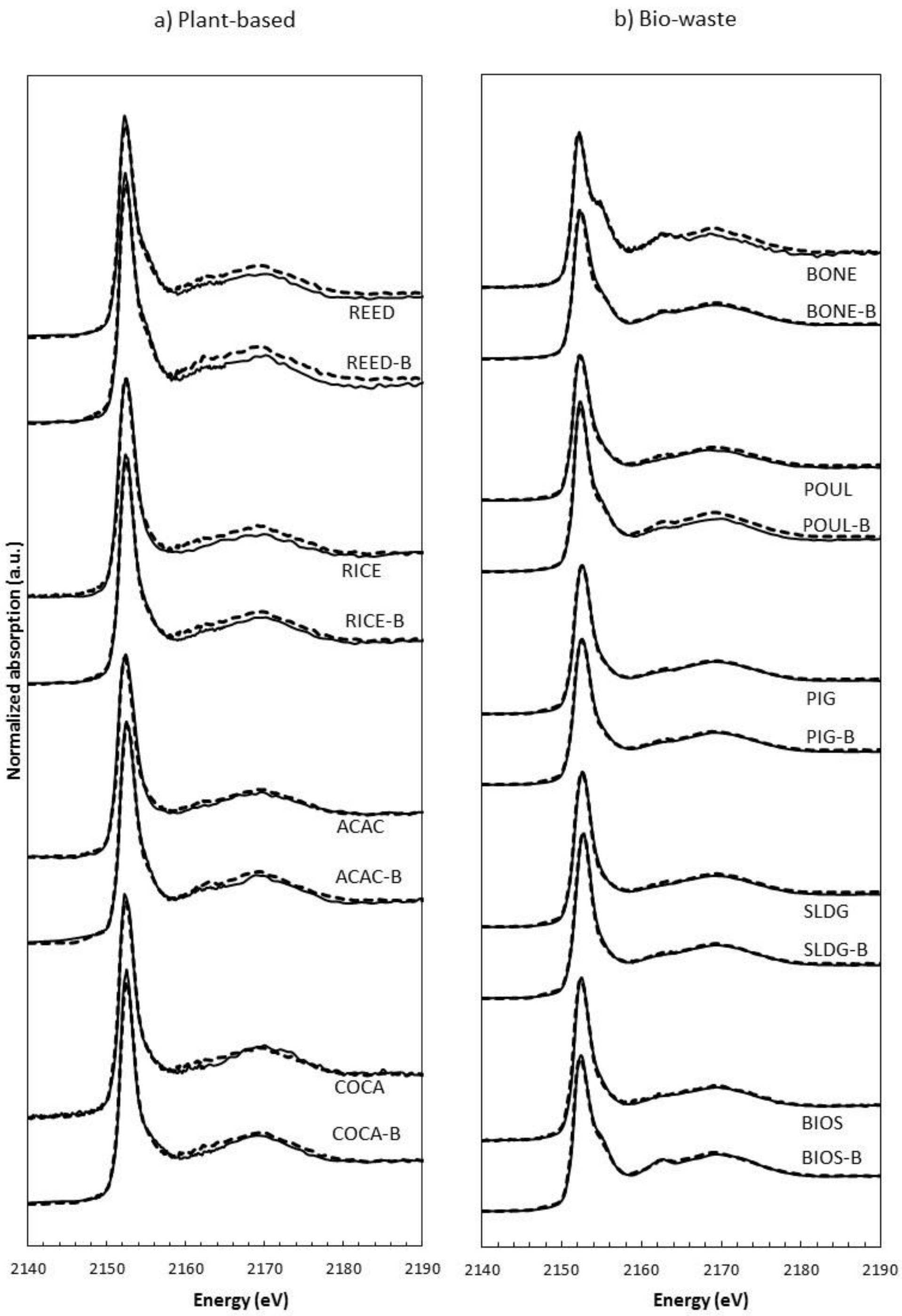

Fig. S1 Stacked normalized P *K*-edge XANES spectra of the biomass feedstocks and their derived biochars (-B): a) plant-based and b) bio-waste materials. Spectra derived from the linear combination fitting of standards are presented as broken lines (------).
